# Study of interface reaction in a B_4_C/Cr mirror at elevated temperature using soft X-ray reflectivity

**DOI:** 10.1107/S1600577522004738

**Published:** 2022-05-25

**Authors:** Mohammed H. Modi, Shruti Gupta, Praveen K. Yadav, Rajkumar Gupta, Aniruddha Bose, Chandrachur Mukherjee, Philippe Jonnard, Mourad Idir

**Affiliations:** a Soft X-ray Applications Laboratory, Raja Ramanna Centre for Advanced Technology, Indore 452013, India; b Homi Bhabha National Institute, Anushakti Nagar, Mumbai 400094, India; c Superconducting Proton Linac Section, Raja Ramanna Centre for Advanced Technology, Indore 452013, India; d Optical Coating Laboratory, Raja Ramanna Centre for Advanced Technology, Indore 452013, India; e Sorbonne Universite, Faculte des Sciences et Ingénierie, UMR CNRS, Laboratoire de Chimie Physique-Matiere et Rayonnement, 4 Place Jussieu, F-75252 Paris Cedex 05, France; f National Synchrotron Light Source II (NSLS-II), Brookhaven National Laboratory, PO Box 5000, Upton, NY 11973, USA

**Keywords:** boron carbide, X-ray mirrors, synchrotron radiation, soft X-ray reflectivity, X-ray optics

## Abstract

Boron carbide is a prominent material for high-brilliance synchrotron optics as it remains stable up to very high temperatures. The present study shows a significant change taking place at 550°C in the buried interface region formed between the Cr adhesive layer and the native oxide layer present on the silicon substrate. An *in situ* annealing study is carried out at the Indus-1 Reflectivity beamline from room temperature to 550°C (100°C steps). The studied sample is a mirror-like boron carbide thin film of 400 Å thickness deposited with an adhesive layer of 20 Å Cr on a silicon substrate. The corresponding changes in the film structure are recorded using angle-dependent soft X-ray reflectivity measurements carried out in the region of the boron *K*-edge after each annealing temperature. Analyses performed using the Parratt recursive formalism reveal that the top boron carbide layer remains intact but interface reactions take place in the buried Cr–SiO_2_ region. After 300°C the Cr layer diffuses towards the substrate. At higher temperatures of 500°C and 550°C the Cr reacts with the native oxide layer and tends to form a low-density compound of chromium oxysilicide (CrSiO_*x*_). Depth profiling of Si and Cr distributions obtained from secondary ion mass spectroscopy measurements corroborate the layer model obtained from the soft X-ray reflectivity analyses. Details of the interface reaction taking place near the substrate region of boron carbide/Cr sample are discussed.

## Abstract 2

Non-destructive interface characterization of boron carbide shows a significant change at 550°C in the interface region formed between an adhesive chromium layer and native oxide layer on silicon substrate, whereas the principal layer of boron carbide remains stable.

## 1. Introduction

Artificial Bragg reflectors (Underwood & Barbee, 1981 ▸), commonly known as multilayer mirrors, have revolutionized the field of X-ray optics particularly for the low-energy X-ray region (below 2000 eV) where natural crystals are available but have severe limitations. To make a good multilayer optics, its constituent layer materials should have good chemical and morphological stability, besides having good refractive index contrast and low absorption in the targeted X-ray energy region. Over the years, different material combinations have been studied and very good reflectivity performances have been achieved. Such multilayer optics have been deployed in various synchrotron beamlines (Kazimirov *et al.*, 2006 ▸; Morawe, 2007 ▸). Many multilayers based on low-*Z* and high-*Z* materials combinations are being used, and among those Mo/Si is one of the most popular multilayers for its high-reflectivity performance in the 100–200 Å wavelength range. Multilayers based on pure elements are prone to degrade early as large differences in the atomic sizes (low-*Z* and high-*Z* atoms) leads to the formation of a rough interface and thereby generates a large volume for intermixing at the interfaces. The recent development of X-ray free-electron laser (XFEL) sources (Allaria *et al.*, 2009 ▸; Ackermann *et al.*, 2007 ▸; Ishikawa *et al.*, 2012 ▸) generating ultra-short X-ray pulses has posed a new challenge for X-ray optics: the need for more stable material combinations for X-ray mirrors. In particular, X-ray pulses of very high intensity induce radiation damage in optical components and therefore numerous research works are being carried out to find a high-stability and high-reflecting material combination for multilayer optics. Multilayers of compound materials have also been proposed to obtain stable performances (Modi, Rai *et al.*, 2012 ▸) in extreme environments.

B_4_C shows very favourable thermal properties, including a high melting point (∼2700°C) and good thermal stability. It is a preferred material for XFEL optical applications. B_4_C-coated Kirckpatrick–Baez (KB) mirrors are used at the Atomic, Molecular and Optical (AMO) science beamline at Linac Coherent Light Source (LCLS) at the SLAC National Accelerator Center (Soufli *et al.*, 2011 ▸). B_4_C is used as a spacer material and also as a diffusion barrier (Wu *et al.*, 2018 ▸; Jonnard *et al.*, 2010 ▸) in multilayer optics in both the soft and hard X-ray regime as it gives smooth and stable interfaces. Jiang *et al.* (2013 ▸) have studied W/B_4_C, La/B_4_C and Mo/B_4_C multilayers to analyse the behaviour of the B_4_C interface with different metal species and found that the metal-on-B_4_C interface is about 2–3 Å thick, while that of the B_4_C-on-metal is >4 Å, which seems reasonable for many applications.

Regarding the performance of B_4_C in extreme conditions, it was reported that at the AMO beamline the KB mirror optics developed a blemish on the mirror surface after about nine months of use, mainly caused by the intense XFEL pulse (Soufli *et al.*, 2011 ▸). Aquila *et al.* (2015 ▸) found a very high single-shot damage threshold for B_4_C. They analysed the single-shot damage threshold fluence of 500 Å-coated B_4_C and ruthenium mirrors on silicon substrate using 7 and 12 keV photons. The Ru showed cracking and ablation above the damage threshold; however, damage in B_4_C was localized to the interface region between B_4_C and the Si substrate. Contrary to other studies, Morawe *et al.* (2020 ▸) reported a major reduction in thickness and composition of a B_4_C(195 Å)/Cr(21 Å)/Si stack after annealing in air at 300°C for 500 minutes. After 700 minutes of annealing in air at 300°C, the B_4_C layer splits into two parts: a top C-rich layer of 100 Å and beneath it a 30 Å B_4_C layer. In transmission electron microscopy measurements, Aquila *et al.* (2015 ▸) showed a sign of interface reaction at the B_4_C/Si interface, with no change in the B_4_C principal layer region. It is therefore desirable to investigate the interface properties of the B_4_C layer at elevated temperatures by applying non-destructive techniques to understand whether an interface reaction is happening at elevated temperatures and also to further establish the stability issue of the B_4_C coating. To the best of our knowledge, interface studies on B_4_C thin films using non-destructive techniques are rather scarce.

In the present study a mirror-like sample of 400 Å-thick boron carbide deposited on a Si substrate with 20 Å adhesive layer of chromium is studied to investigate the stability of the boron carbide principal layer and related modifications at the interfaces at elevated temperatures up to 550°C at the Indus-1 reflectivity beamline. It is found that the principal boron carbide layer remains intact; however, the interface region near the Cr adhesive layer and the native oxide layer on the Si substrate undergoes a significant change as Cr diffuses towards the substrate and tends to form a low-density compound of chromium oxysilicide.

## 2. Experimental

### 2.1. Sample preparation

A 400 Å-thick boron carbide mirror-like sample was deposited on a silicon substrate with a 20 Å adhesive chromium layer using a pulsed DC sputtering system. Prior to deposition, the substrate was ultrasonically cleaned. A commercially available 4-inch-diameter B_4_C target of 99.99% purity was used for deposition and argon gas of 99.99999% purity was used as a sputtering medium. Before the deposition process, a base pressure in the chamber of ∼5 × 10^−7^ mbar was obtained. With Ar at 40 sccm (standard cubic centimetre per minute) flow rate, a pressure of ∼3 × 10^−3^ mbar was maintained in the chamber. Film thickness and deposition rate were monitored using a quartz crystal thickness monitor.

### 2.2. Characterizations

For the structural characterization of the thin film, grazing-incident X-ray reflectivity (GIXRR) measurements were performed using a Bruker D-8 system consisting of a θ–2θ goniometer and a copper *K*_α_ hard X-ray source (1.54 Å). The angular step size for the θ–2θ scan was kept as 0.005° in order to observe small variations in the interference fringes.

Angle-dependent soft X-ray reflectivity (SXR) measurements and *in situ* annealing experiments have been carried out using the Reflectivity beamline, BL-04 (Nandedkar *et al.*, 2002 ▸), at the Indus-1 synchrotron radiation facility. The main optical components of the Reflectivity beamline are a grazing-incidence toroidal grating monochromator and toroidal mirrors for pre- and post-focusing optics. The beamline is designed to cover the 10–300 eV photon energy range. Different harmonic suppression edge filters are installed in the beamline to reduce higher harmonic contamination (Modi, Gupta *et al.*, 2012 ▸). Further details of the beamline are given by Nandedkar *et al.* (2002 ▸).

The experimental station in this beamline is a high-vacuum reflectometer (Lodha *et al.*, 2004 ▸). The goniometer assembly of the reflectometer station comprises two rotary stages and one linear stage. The two rotary stages can be moved in coupled and uncoupled mode to carry out different modes of reflectivity, *i.e.* detector scan, rocking curve scan, θ–2θ scan *etc*. The linear stage is employed to bring the sample in and out of the direct beam, to facilitate direct beam monitoring. A silicon XUV photodiode is used to monitor the reflected beam intensity. To perform *in situ* annealing experiments, a sample heater made of boron nitride is mounted on the sample stage with custom-designed housing.

Energy-dependent SXR measurements near the boron *K*-edge (188 eV) were carried out using the Soft X-ray Reflectivity beamline, BL-03 (Modi *et al.*, 2019 ▸), of the Indus-2 synchrotron source. The beamline is equipped with a varied-line-spacing plane-grating monochromator (VLS-PGM) which operates in constant deviation angle geometry. The beamline provides photons in the energy range 100–1500 eV with moderate resolving power of 1000–6000 using three gratings with line densities of 150, 400 and 1200 lines mm^−1^. The beamline operating in an ultra-high-vacuum environment is separated from the experimental station working in ∼10^−7^ mbar using a custom-designed differential pumping system. The reflectivity measurements were performed in *s*-polarization geometry with the goniometer working in a vertical dispersion geometry. The reflected beam from the sample is measured using an Si photodiode detector (International Radiation Detectors Inc., USA) with 100% internal quantum efficiency.

To improve the spectral purity of the monochromatic light coming from the VLS-PGM, a three-mirror-based higher-order suppressor (HOS) was used. The three mirrors have a stripe coating of four different materials, *i.e.* carbon, chromium, silicon and nickel. In order to efficiently suppress the higher harmonics in the 60–225 eV range a stripe of carbon coating is used. During the operation of the HOS the three mirrors can be set in the beam path with similar coatings on respective mirrors. The HOS setup improves the spectral purity and reduces the harmonic components below 0.1%.

Secondary ion mass spectroscopy (SIMS) measurements were performed using a Cs^+^ ion gun operating at 1 keV (TOFSIMS-5, IONTOF) to sputter the sample. However, the analysis was carried out using Bi^+^ ions operating at 30 keV, 4.7 pA. The analysis area was 100 µm × 100 µm inside the sputter crater of 300 µm × 300 µm.

The Parratt recursive formalism (Parratt, 1954 ▸) was used for the analysis of the measured reflectivity data. The surface roughness effect was taken into account using the Névot–Croce model (Névot & Croce, 1980 ▸). The fitting of the experimental data is carried out using the *Labview*-based *SRxrr* tool (Modi *et al.*, 2008 ▸). This tool has many unique features as it considers higher-harmonic effects, polarization factors, beam divergence and background noise. This tool calculates reflectivity and transmission both as a function of angle and photon energy/wavelength.

## 3. Results and discussions

**Figure 1:**
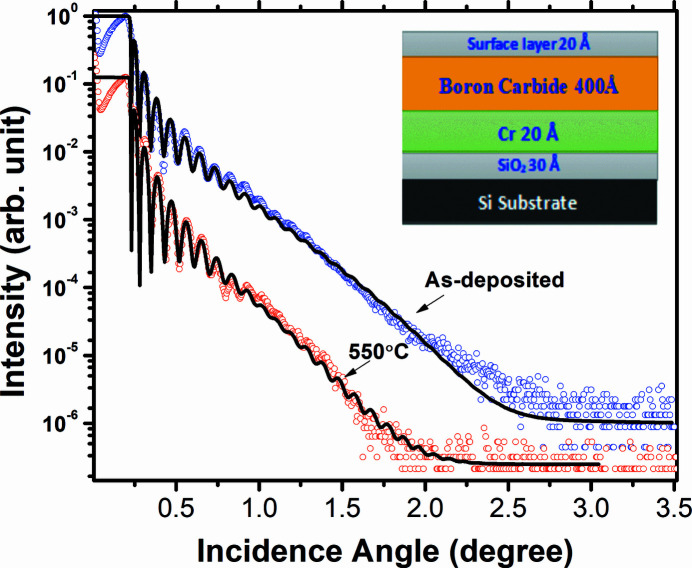
Measured (open circles) and fitted (continuous line) GIXRR curves of as-deposited and 550°C annealed boron carbide/Cr bilayer samples. The layer model used to obtain the best fit is also shown. For the sake of clarity, the curves are vertically shifted.

**Figure 2:**
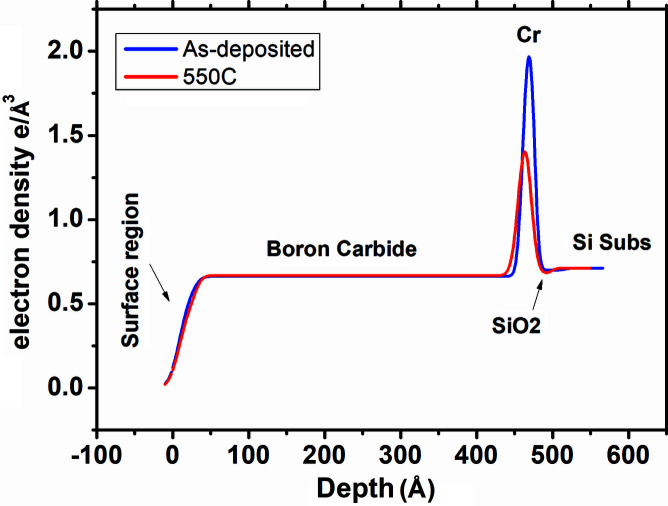
Electron density profile of as-deposited and 550°C annealed boron carbide/Cr bilayer samples as obtained from the GIXRR fit.

Fig. 1 ▸ shows the measured and fitted GIXRR curves of 400 Å-thick boron carbide thin film. To obtain a best fit of the GIXRR data, various models were tried but the best fit was obtained using a four-layer model which is shown in the inset of Fig. 1 ▸. The model consists of a native oxide layer on the Si substrate, a thin Cr layer as used for improving the film adhesion, a principal boron carbide layer and a top surface layer arising due to interaction of the surface with the ambient. The values of the thickness, roughness and real part of the refractive index (δ) of the native oxide layer, Cr layer, principal boron carbide layer and top surface layer as obtained from the best fit of the as-deposited sample are given in Table 1 ▸. The electron density profile (EDP) (Banerjee *et al.*, 2004 ▸) of the sample as obtained from the GIXRR analyses is plotted in Fig. 2 ▸, which represents the density variation across the depth of the sample. It is evident from the EDP that the boron carbide thin film is uniform across the depth except near the air/film region where it forms a low-density surface layer due to reaction with the ambient. Near the film/substrate region a peak in the EDP corresponds to a thin Cr layer which was deposited for improving adhesion with the silicon substrate. The thickness of the Cr layer is found to be 15.7 Å which is slightly less than the targeted thickness value of 20 Å. A tiny dip in the film/substrate region as indicated by an arrow corresponds to the native oxide layer. Since the delta value of bulk SiO_2_ is 7.122 × 10^−6^ which is slightly less than the delta value of bulk Si (7.577 × 10^−6^), this difference gives rise to a dip in the density profile. In the present case of the as-deposited sample this dip is not prominent because the fit value of the SiO_2_ delta is found to be slightly higher by ∼4.4% (7.453 × 10^−6^). For the as-prepared sample the density of the boron carbide layer is found to be 2.33 g cm^−3^ which is 91.7% of the bulk density (2.54 g cm^−3^).

In order to analyze the behaviour of the interfaces in the boron carbide/Cr bilayer at elevated temperatures, the same sample was annealed up to 550°C in steps of 100°C for 2 h at each temperature. The annealing experiments were carried out at the reflectometer station at the Indus-1 Reflectivity beamline in a ∼1 × 10^−6^ mbar vacuum. After each annealing experiment, angle-dependent SXR data were recorded *in situ* as discussed in the next section. The in-vacuum heater assembly mounted on the goniometer supports temperatures up to 550°C. Thereafter the sample was taken out and further characterized via GIXRR measurement.

The measured and best-fitted GIXRR curves of the 550°C annealed sample are shown in Fig. 1 ▸. The parameters of the best-fitted curve of the annealed sample are mentioned in Table 2 ▸. After the 550°C annealing the boron carbide layer thickness slightly decreased from 431 Å to 428 Å (∼0.6% drop) whereas its density increased in a similar ratio from 2.33 g cm^−3^ (91.7% of the bulk value) to 2.35 g cm^−3^ (92.5% of the bulk value). The roughness of the principal boron carbide layer does not change much – it reduced from 8.0 Å to 7.0 Å. After the annealing, the electron density profile as shown in Fig. 2 ▸ indicates that a major change is taking place near the Cr film region. The effective electron density of the Cr layer is reduced from 1.97 e^−^ Å^−3^ (as-deposited) to 1.4 e^−^ Å^−3^ (550°C annealed sample). After the annealing at 550°C, the thickness and the density of the boron carbide layer have not changed much. The EDP suggests a major change in the Cr layer region which is likely due to interaction between the Cr layer with the native silicon oxide layer. The GIXRR technique is less sensitive to any changes taking place in the SiO_2_ layer because of the poor refractive index contrast between Si and SiO_2_. Therefore to gain more insight about the changes taking place in the SiO_2_ layer due to its interaction with the Cr layer, the *in situ* SXR data taken during the annealing experiments are analyzed, as discussed in the next section.

### 3.1. Soft X-ray reflectivity

**Figure 3:**
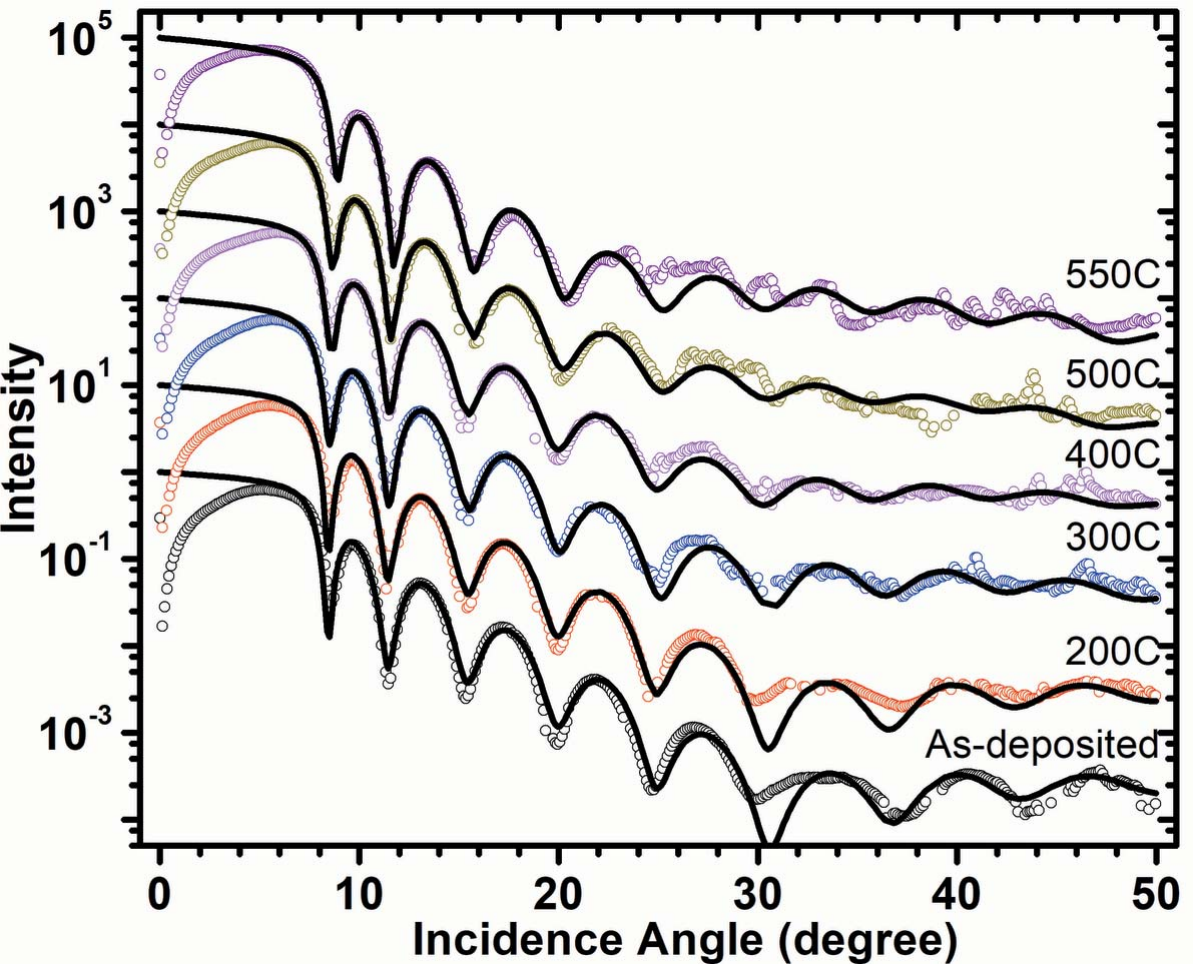
Measured (open circles) and fitted (continuous line) soft X-ray reflectivity curves of as-deposited and annealed samples using 155 eV incident photon energy (80 Å).

**Figure 4:**
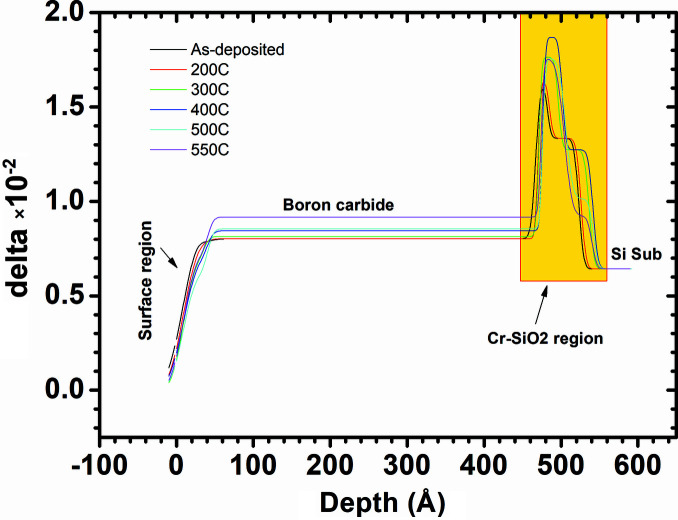
Optical density profile obtained from the best fit of SXR data taken after each annealing temperature. The region marked in the box is zoomed and shown in Fig. 6 ▸.

**Figure 5:**
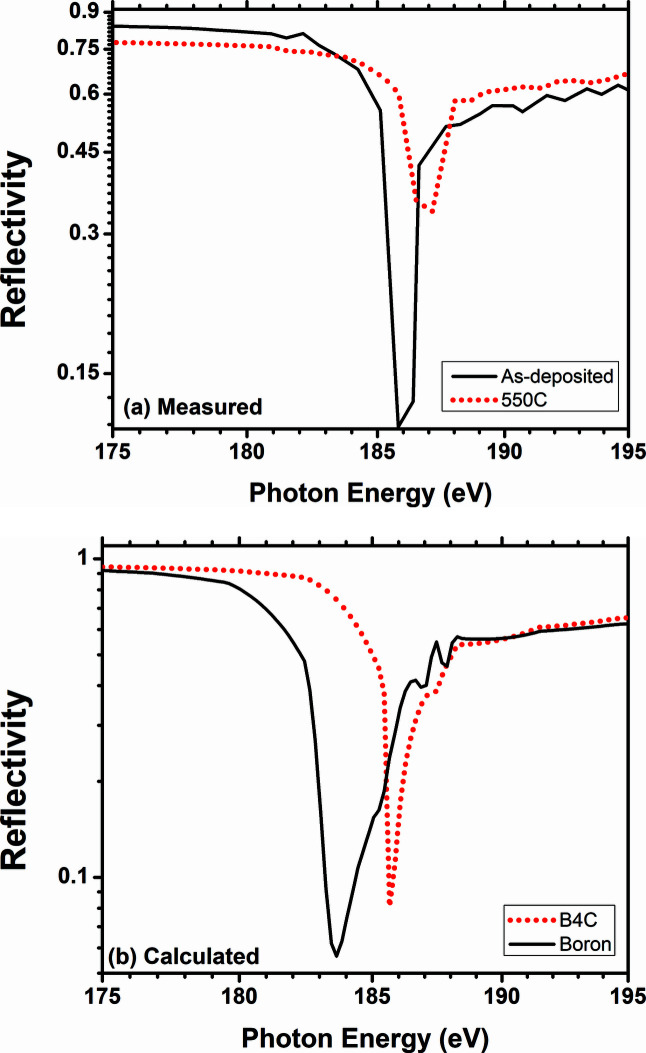
(*a*) Measured energy-dependent soft X-ray reflectivity of the boron carbide/Cr bilayer at 1.5° grazing-incidence angle for the as-deposited and 550°C annealed sample. (*b*) The calculated reflectivity of stoichiometric B_4_C and boron is compared.

Angle-dependent SXR data measured after each annealing temperature are shown in Fig. 3 ▸. The boron carbide/Cr bilayer shows distinct interference fringes up to 50° glancing angle. The fringe pattern has a good contrast up to 550°C annealing temperature. These SXR data are analyzed using the same structural model as used for GIXRR analyses. For as-deposited sample the structural parameters obtained from the GIXRR analyses are used and kept fixed while the SXR fitting and only the δ (refraction) and β (absorption) parameters are varied to obtain a best fit. For high temperature, the structural parameters (thickness, roughness) are also considered as fitting variables in order to account for any changes happening in the film structure at the elevated temperatures. For the SXR data of 550°C, again structural parameters as obtained from the GIXRR analyses of 550°C annealed sample are used and only δ and β are varied. After a rigorous SXR data fitting the final optical density profiles (delta profile) of the boron carbide/Cr bilayer for each temperature are obtained and plotted in Fig. 4 ▸. Contrary to the EDP profile obtained from the GIXRR data (Fig. 2 ▸), the SXR analyses suggest a clear change taking place near the Cr–SiO_2_ region upon annealing along with some minor change in the principal film region. Optical density of the boron carbide layer is increased by ∼14% whereas its layer thickness is marginally decreased by 2 Å (<0.5%) on annealing from room temperature to 550°C. Both GIXRR and SXR analyses confirm a 2–3 Å decrease in the principal boron carbide layer thickness after the annealing. The SXR analyses show a gradual increase in the delta values of the boron carbide layer from 8.02 × 10^−3^ (as-deposited sample) to 9.17 × 10^−3^ (550°C annealed sample). The delta value of the as-deposited sample is ∼20% lower than the stoichiometric bulk boron carbide value of 1.02 × 10^−2^, and for the 550°C annealed sample it is ∼10% lower. The increase in the delta value of the principal boron carbide layer upon annealing suggests the thin film is becoming more stoichiometric with annealing. The change in stoichiometry of the principal layer is further confirmed from the energy-dependent SXR data measured near the boron *K*-edge region in which an edge shift of ∼1.0 eV towards higher energy is observed after the annealing at 550°C [see Fig. 5 ▸(*a*)].

The energy-dependent SXR data as measured before and after the annealing at fixed 1.5° glancing incidence angle are shown in Fig. 5 ▸(*a*). A prominent dip in the reflectivity spectra of the pristine sample is observed at 186 eV whereas for the annealed sample it is shifted near 187 eV. In the case of the as-deposited sample the dip is strong indicating the film is boron rich as higher boron content causes increased absorption near its *K*-edge region that in turn lowers the reflectivity value. The calculated curve of boron is compared with that of B_4_C in Fig. 5 ▸(*b*) where it is obvious that the dip is prominent in the boron case and shifts towards higher energy for boron carbide. In the literature the reported *K*-edge value of bulk boron is 188 eV (Henke *et al.*, 1993 ▸) whereas for stoichiometric B_4_C the reported value of the edge lies in the 189–191.6 eV range (Jiménez *et al.*, 1999 ▸; Feng *et al.*, 2021 ▸). In X-ray absorption measurements performed on boron-rich carbide film (B_11_C), Jimenez *et al.* (1999 ▸) observed the boron *K*-edge at 187 eV. In the present case of 400 Å-thick boron carbide film the position shifts of ∼1.0 eV towards higher energy upon annealing at 550°C indicate an increase in the stoichiometric nature of the pulsed DC sputtering grown boron carbide thin film.

**Figure 6:**
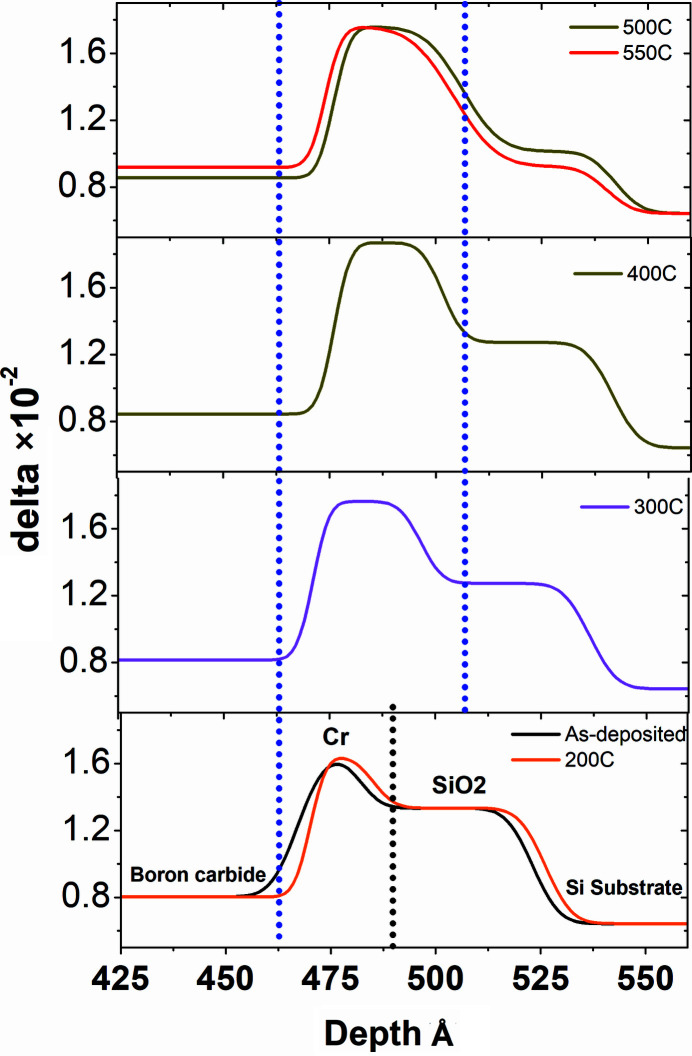
The optical density profile of the Cr–SiO_2_ region is plotted for each annealing temperature.

Coming back to Fig. 4 ▸ where the Cr–SiO_2_ region shows an interesting change upon annealing, this region is zoomed in Fig. 6 ▸ and curves are plotted separately for each annealing temperature. At 200°C, the Cr–SiO_2_ region does not change much; however, at 300°C annealing the thickness of the Cr region increases from 15 Å to 25 Å whereas the thickness of the SiO_2_ region remains unchanged at 40 Å. The increase in thickness of the Cr region suggests a possible swelling of the Cr layer due to oxidation as it interacts with the nearby native oxide layer. Since the density of bulk Cr is 7.19 g cm^−3^ and that of bulk Cr_2_O_3_ is 5.22 g cm^−3^, oxidation of Cr atoms causes a decrease in film density and that in turn increases the film thickness. At higher temperatures (>400°C) the probability of Cr interaction with Si and oxygen atoms to form an oxysilicide phase increases as indicated from the decrease in the delta value of the Cr region for 500°C and 550°C temperature with respect to that of the 400°C temperature value. For an 80 Å probing wavelength the delta value of bulk Cr is 2.84 × 10^−2^ and for bulk CrSi_2_ is 0.61 × 10^−2^. Kuznetsova *et al.* (2021 ▸) have reported the formation of CrSi_2_ at the interface of 300 Å-thick Cr film on silicon substrate after rapid thermal annealing at >400°C. Furthermore, the delta value of the SiO_2_ region decreases from 1.33 × 10^−2^ to 9.23 × 10^−3^ and it is tending towards a Si delta value of 6.42 × 10^−3^. This indicates that the native oxide layer is shrinking upon annealing.

**Figure 7:**
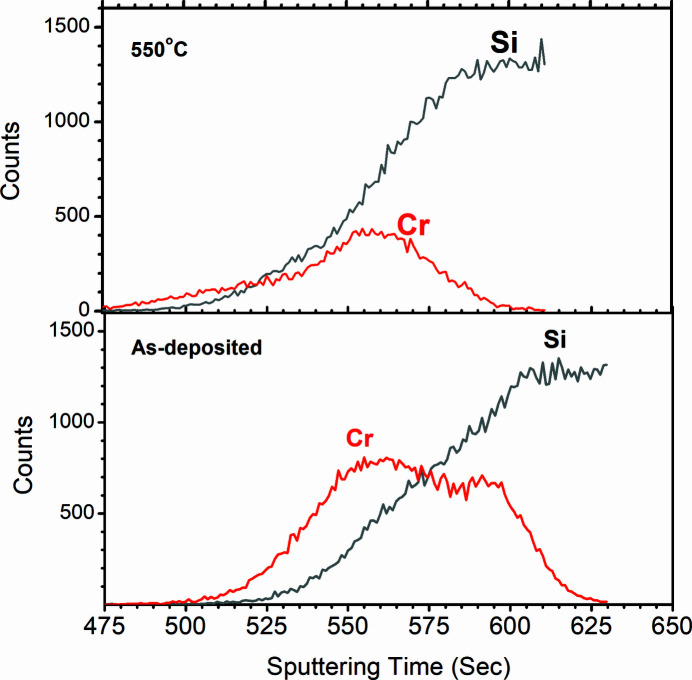
Cr and Si distributions in the bilayer sample as obtained as a function of sputtering time from SIMS measurement. Only the Cr–SiO_2_ region is shown.

To corroborate the interface model deduced from the SXR analyses, SIMS measurements were carried out on as-deposited and 550°C annealed sample. The SIMS data confirm no change in the boron carbide principal layer upon annealing. The SIMS profile of the Cr–SiO_2_ region is plotted in Fig. 7 ▸, where the distributions of the Cr and Si atoms are shown as a function of sputtering time. In the as-deposited sample the Cr signal starts after 500 s and Si starts after 525 s. The Si signal rises steadily and reaches a constant level once it enters the substrate region. Therefore, the steadily rising Si signal is due to the native oxide layer and then the flat region is due to the Si substrate. The distribution of Cr shows its spread in a wide region where some Cr lies before the native oxide layer and some Cr inside the native oxide region. After annealing, the SIMS data indicate that the Cr atoms diffuse inside the native oxide layer, as is also found in the SXR analyses. The major portion of Cr lies inside the native oxide region where it is likely to form an oxysilicide phase as no independent Cr layer exists after the 550°C annealing as per the SIMS measurement. The SIMS data corroborate the SXR model indicating diffusion of Cr towards the native oxide region. Contrary to the SXR model the SIMS measurement shows a shrinkage in the Cr region upon annealing. This could be due to variation in sputtering yields of Cr and CrSiO_*x*_. Beside this contradiction, both SIMS and SXR analyses clearly indicate an interface reaction in the Cr–SiO_2_ region in boron carbide mirror-like sample where Cr diffuses towards the substrate upon annealing at 550°C. The GIXRR analysis was able to indicate a reduction in film density in the Cr region after 550°C annealing, whose details could be unfolded by soft X-ray reflectivity analyses.

## 4. Conclusions

A mirror-like sample of 400 Å-thick boron carbide thin film is deposited with a 20 Å Cr adhesive layer on a silicon substrate and is studied for interface reaction by performing *in situ* annealing experiments. The sample is annealed *in situ* from room temperature to 550°C in steps of 100°C for 2 h at each temperature. GIXRR, SXR and SIMS data revealed that the boron carbide layer is stable and its structural properties are not affected by the annealing. However, a significant change is observed at the Cr/substrate interface region due to reaction of the Cr layer with the native oxide layer present on the silicon substrate. The GIXRR measurements indicated a reduction in Cr density upon annealing, whose details could be unfolded by SXR analyses. SXR analyses showed a diffusion of Cr towards the Si substrate and the formation of a low-density compound of chromium oxysilicide due to reaction of Cr with Si at the elevated temperature. SXR could reveal subtle details about the evolution of the buried interfaces beneath the 400 Å-thick boron carbide layer whose results are corroborated by SIMS measurements.

The SXR analyses give a clear insight about the interface reaction at the buried Cr/SiO_2_ interface in a non-destructive manner. Such a buried interface lying underneath a thick film of 400 Å is very difficult to analyze by other means as other techniques primarily depend on sequential sputtering and subsequent measurement of sputtered species. X-ray photoelectron spectroscopy and secondary ion mass spectroscopy have such limitations and also pose certain artefacts coming from the sputtering process. Also converting sputtering time to depth is difficult because of the large variation in sputtering yield among different elements.

## Figures and Tables

**Table 1:** GIXRR fit parameters of boron carbide/Cr as-deposited sample

Layer	Thickness (Å)	Roughness (Å)	δ
Surface layer	26	11.0	6.110 × 10^−6^
Boron carbide	431	8.0	7.063 × 10^−6^
Cr	15.7	5.7	2.303 × 10^−5^
SiO_2_	40.0	4.6	7.453 × 10^−6^
Si substrate–	–	5.0	7.577 × 10^−6^

**Table 2:** GIXRR fit parameters of boron carbide/Cr after 550°C annealing

Layer	Thickness (Å)	Roughness (Å)	δ
Surface layer	21.6	11.0	5.410 × 10^−6^
Boron carbide	428.0	7.0	7.103 × 10^−6^
Cr	10.7	8.7	2.303 × 10^−5^
SiO_2_	30	7.5	7.253 × 10^−6^
Si substrate	–	5.0	7.577 × 10^−6^
